# Behavioural and Psychological Symptoms in Poststroke Vascular Cognitive Impairment

**DOI:** 10.1155/2014/430128

**Published:** 2014-03-02

**Authors:** Meena Gupta, Abhijit Dasgupta, Geeta Anjum Khwaja, Debashish Chowdhury, Yogesh Patidar, Amit Batra

**Affiliations:** Department of Neurology, GB Pant Hospital, Jawaharlal Nehru Road, New Delhi 110002, India

## Abstract

*Background.* Behavioural and psychological symptoms of dementia (BPSD) cause significant patient and caregiver morbidity in vascular cognitive impairment (VCI). *Objectives*. To study and compare the occurrence and severity of BPSD between multi-infarct dementia (MID), subcortical ischaemic vascular disease (SIVD), and strategic infarct subtypes of poststroke VCI and to evaluate the relationship of these symptoms with the severity of cognitive impairment. *Methods*. Sixty patients with poststroke VCI were classified into MID, SIVD, and strategic infarct subtypes. BPSD were studied by the neuropsychiatric inventory (NPI). The severity of cognitive impairment was evaluated by the clinical dementia rating scale (CDR). *Results*. 95% of cases had at least one neuropsychiatric symptom, with depression being the commonest, irrespective of subtype or severity of VCI. Strategic infarct patients had the lowest frequency of all symptoms. SIVD showed a higher frequency and severity of apathy and higher total NPI scores, compared to MID. Apathy and appetite disturbances occurred more commonly with increasing CDR scores. The total NPI score correlated positively with the CDR score. *Conclusion*. Depression was the commonest neuropsychiatric symptom in VCI. The neuropsychiatric profiles of MID and SIVD were similar. The frequency and severity of apathy and the net burden of BPSD increased with increasing cognitive impairment.

## 1. Introduction

Behavioural and psychological symptoms of dementia (BPSD) form an integral part of the natural history of dementia. Dementia patients develop changes in mood, personality, perception, thought, and vegetative functions like sleep and appetite, which affect the daily functions of the patient and are potentially disruptive to the family. The causes of BPSD are far from clear. These symptoms may arise due to perturbations in the neurotransmitter milieu that underlie the pathogenesis of dementia. Environmental factors also influence the occurrence of BPSD. Dementia results in greater susceptibility to environmental stimuli and a lower threshold at which these stimuli affect behaviour. People with dementia progressively lose their coping abilities, leading to the manifestation of various behavioural abnormalities.

BPSD have been found to lead to a more rapid cognitive decline in patients with dementia [1] and to correlate negatively with their survival [2]. They also contribute significantly to caregiver distress. Caregivers of dementia patients report BPSD, especially symptoms like aggression and screaming, to be the most difficult problem to cope with [3]. The management of BPSD has the potential to alleviate much of the suffering of dementia patients and their caregivers. Notwithstanding the importance of these symptoms, there has traditionally been an overbearing emphasis on cognitive dysfunction alone in the classical descriptions of most dementia subtypes.

Vascular cognitive impairment (VCI) as currently understood refers to the entire spectrum of cognitive impairment occurring as a result of cerebrovascular disease. The present concept of VCI encompasses not only vascular dementia (VaD), but also mixed dementia and vascular cognitive impairment-no dementia (VCI-ND). VCI-ND refers to that subgroup of patients who manifest cognitive deficits resulting from cerebrovascular disease, but do not meet the definition of dementia. As compared to Alzheimer disease, the literature dealing with BPSD in VaD is relatively modest, especially so in the context of the modern concept of VCI. The present study aimed to examine the profile of neuropsychiatric symptoms in VCI.

## 2. Aims of the Study

To study the behavioural and psychological symptoms in patients with poststroke VCI and their relationship with the subtype and severity of VCI.

## 3. Methods

This prospective, observational study was approved by the institutional ethics committee. All patients who presented to our Neurology Department between March 2011 and August 2012 at least 3 months after a stroke were interrogated regarding the occurrence of cognitive dysfunction in the presence of a reliable caregiver. Those who were found to have developed cognitive deficits following a stroke, scored ≥4 on the Hachinski Ischaemic Score [4], and gave written and informed consent to participate in the study were made to undergo a detailed neurological and mental status examination. Cognitive domains like language, attention, executive function, memory, visuospatial function, praxis, and calculation were clinically evaluated using appropriate tests. Patients who had aphasia severe enough to impair the evaluation of other cognitive domains, those with clinical features of degenerative dementias like Alzheimer's disease, dementia with Lewy bodies, and frontotemporal lobar degeneration, those with hepatic, renal, or thyroid dysfunction, and those who were already on antidementia drugs, cognition enhancers, antidepressants, anxiolytics, or antipsychotics were excluded. All patients underwent a brain MRI scan (1.5 or 3 Tesla, incorporating T1, T2, FLAIR, diffusion-weighted images, ADC maps, and GRE sequences.). Vascular dementia was diagnosed in cases fulfilling the NINDS-AIREN criteria for probable VaD [5]. Cases consistent with the criteria described by the Canadian Study of Health and Ageing were designated vascular cognitive impairment-no dementia (VCI-ND) [6]. VCI comprises both the above subsets of patients. Based on the MRI findings, cases were classified into multi-infarct dementia (MID) (multiple cortical-based infarcts), strategic infarct (strategic) (a single infarct in a cortical or subcortical location correlating with the clinical localisation of the observed deficits on higher mental function assessment), and subcortical ischaemic vascular disease subgroups (SIVD) (multiple lacunar infarcts and/or subcortical T2 hyperintensities involving ≥25% of white matter). Cases with mixed multi-infarct dementia and SIVD features on the MRI and those with evidence of intracerebral haemorrhage or venous sinus thrombosis were excluded. The clinical dementia rating scale (CDR) score [7] was used to determine the severity of cognitive impairment.

BPSD were studied on the neuropsychiatric inventory (NPI) [8] based on an interview with a responsible caregiver. For each of the 12 symptoms, the product of the frequency and severity scores was recorded as the domain score. Based on the sum of the domain scores, a total NPI score for each patient was calculated.

### 3.1. Statistical Analysis

Mean age and educational status were compared between the MID, SIVD, and strategic infarct groups by the one-way analysis of variance (ANOVA). The sex distribution between these groups was studied by means of the Chi-square test. The differences in the frequency of various symptoms between MID, SIVD, and strategic infarct groups and between the 4 classes based on the CDR were also studied by means of the Chi-square test. The differences in individual NPI domain scores and the total NPI scores between MID and SIVD groups were studied by means of the Mann-Whitney *U* test. Correlations between the CDR score and individual domain scores or the total NPI score were estimated by Spearman's correlation coefficients. (*ρ*) A *P*  value ≤ 0.05 was considered to be statistically significant.

## 4. Results

A total of 60 patients of VCI were included. Overall, the mean age was 58.8 (±12.03) years and the male : female ratio was 2 : 1. The mean number of years of education was 8 (±5.14). MID, SIVD, and strategic subtypes constituted 25, 21, and 14 patients, respectively. There was no significant difference regarding age, sex, and educational status between the three subtypes (Table 1). The CDR scores ranged from 0.5 to 3. Based on the CDR scores, the MID and SIVD groups were comparable. Most strategic cases had a CDR score of 0.5.

### 4.1. Frequency of Various BPSD (Table 2, Figure 1)

At least one behavioural or psychological symptom on the NPI was found in 57 out of 60 cases (95%). 3 cases (5%) had no BPSD. Overall, the mean number of symptoms was 3.98 (±2.11). Depression was the commonest BPSD at 73.3%, followed by appetite disturbances (65%), irritability (51.6%), and anxiety (41.6%). Apathy was found in 35% of the cases. Disinhibition (8%), euphoria, and hallucinations (5% each) were the least common of the BPSD on the NPI. Comparing the frequency of individual symptoms between the four CDR score groups (i.e., CDR = 0.5, 1, 2, and 3), the frequency of both apathy and appetite disturbances was found to differ significantly between the CDR groups and showed an increase with increasing CDR scores (*P* = 0.001 for each). Euphoria occurred only in the CDR 0.5 and 1 groups, though the difference was not statistically significant.

The profile of BPSD in the three subtypes of VCI was compared. Depression was found to be the commonest symptom irrespective of the subtype. The frequency of each symptom was the lowest in the strategic group. Each of the 3 cases who did not manifest BPSD belonged to the strategic infarct group, with the infarcts being in the left angular gyrus, right putamen, and right basifrontal region, respectively. Tests of statistical significance were applied only between MID and SIVD groups as they were comparable on the basis of the CDR scores. These two groups differed only in the domain of apathy which was significantly commoner in SIVD (*P* = 0.002). Euphoria was seen only in the MID subgroup, though the difference was not significant.

### 4.2. Severity of BPSD

Based on the NPI subscale scores, depression was found to be the most severe symptom, followed by irritability, appetite disturbances, apathy, and agitation/aggression (mean subscale scores 5.63, 3.7, 3.35, 3.083, and 3.08, resp.). The lowest mean score was seen in the domain of hallucinations (0.08). The apathy scores showed a robust correlation with the CDR score (*r* = 0.72, *P* < 0.0001). Significant positive correlations were also observed in the domains of night-time behaviour disturbances (*r* = 0.58, *P* < 0.0001) and appetite disturbances (*r* = 0.49, *P* = 0.0002). Correlation was insignificant in the anxiety (*r* = 0.18, *P* = 0.15), depression (*r* = 0.2, *P* = 0.07), and the other remaining domains. A significant positive correlation existed between the total NPI score and the CDR score (*r* = 0.598, *P* = 0.001).

**Figure 2:**
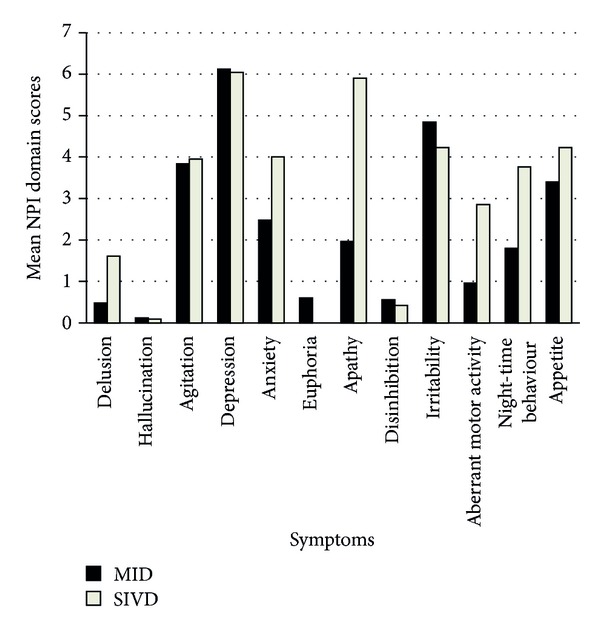
Comparison of the mean NPI subscale score of each symptom between MID and SIVD groups. Note: though the mean values have been depicted, statistical analysis was done by using the Mann-Whitney *U* test for nonparametric data.

Comparing between MID and SIVD groups, the SIVD group had a higher severity of delusions, agitation/aggression, anxiety, apathy, aberrant motor activity, and night-time behaviour and appetite disturbances as compared to MID (Figure 2); however, the difference was statistically significant only for apathy (*P* = 0.01). The SIVD group had a significantly higher total NPI score (mean (SD) score: MID versus SIVD; 26.92 (12.01) versus 37.14 (17.21), median score MID versus SIVD; 27 versus 36 (*P* = 0.049)).

## 5. Discussion

Neuropsychiatric symptoms are important noncognitive features of VCI. These symptoms have been described to be compatible with a diagnosis of vascular dementia as per the Hachinski Ischaemic Score and the NINDS-AIREN criteria. There is a dearth of studies documenting the profile of BPSD in VaD and more so in the broader clinical construct of VCI.

The occurrence of BPSD in our study was 95%, which was similar to other previous studies of BPSD in VaD and VCI [9, 10]. We found depression to be the commonest symptom, as described previously by Srikanth et al. [11]. On the other hand, Staekenborg et al. [9] and Fuh et al. [12] reported apathy to be the commonest neuropsychiatric symptom in VaD. Euphoria was the least common symptom in our study, which was in well agreement with previous studies [9, 13]. Most earlier studies dealt only with VaD. On the other hand, Chiu et al. studied patients with VCI with or without dementia and found sleep disturbances and depression to be the commonest symptoms [10]. The pattern of BPSD may differ depending on the cohort studied, the definition of VCI/VaD, and the instrument used to study the symptoms. All symptoms were less common in the strategic group as compared to MID and SIVD groups, which could probably be explained on the basis of the fact that these patients had lower CDR scores. The profile of BPSD in the MID and SIVD groups was comparable, except a significantly higher occurrence and severity of apathy in SIVD. Apathy is known to be common in subcortical ischaemic vascular disease owing to the occurrence of white matter lesions and/or lacunar infarcts in the basal ganglia and thalami, which lead to interruption of corticosubcortical circuits. Apathy has been variously described to be commoner and more severe in SIVD [9] and in MID [12]. Though not statistically significant, we found a higher frequency of hallucinations, agitation/aggression, irritability, and euphoria in our MID group in good agreement with other studies. Aggression [9, 12], euphoria [9], and irritability [9] have been reported to be commoner in MID in other studies as well. Aggressive behaviour is common in stroke, especially in the territories of the large cerebral vessels [14]. Recently, there has been an attempt to group the BPSD into symptom clusters depending on the frequency of their cooccurrence and to hypothesise a similar pathogenic mechanism for each symptom in a cluster. Irritability, euphoria, and agitation, among others, have been grouped into the “hyperactivity” cluster by Aalten et al. [15]. It is interesting to note on the basis of our findings and those of a previous study [9] that MID patients are more likely to display these “hyperactivity” symptoms, whereas apathy, appetite, and eating disturbances which are part of the “apathy” cluster are commoner in SIVD.

Overall, the total NPI score was found to be higher in SIVD than in MID as compared to previous studies which reported no difference between the groups [9] or a higher score in MID [12]. The total NPI score demonstrated a good positive correlation with the CDR score. Another study also reported a positive correlation between quantitative BPSD scores and the severity of cognitive impairment in VaD [16].

Separately studying the correlation of the severity of individual symptoms with the CDR score, the apathy score was found to correlate most robustly with the severity of cognitive impairment in VCI, followed by appetite and night-time behaviour disturbances. Anxiety and depression scores showed insignificant correlation with the CDR score. Few studies have attempted to correlate the severity of individual symptoms with the degree of cognitive impairment. Aharon-Peretz et al. showed a positive correlation of CDR with appetite and night-time behaviour scores but did not find significant correlations with apathy, anxiety, depression, or euphoria in their cohort of subcortical VaD [17]. Increasing vascular lesion burden in the brain may contribute to worsening cognitive and behavioural performance in patients with VCI. However, lesion volume and lesion location may show differential effects on cognition and behaviour. Hence, there may not be a simplistic linear correlation between cognitive and behavioural disturbances in VCI.

The strengths of our study are the inclusion of patients of VCI with and without dementia, the observation of BPSD symptoms in strategic infarcts, and the attempt to study BPSD with respect to both the subtype and severity of VCI. However, the relatively small sample size is a major limitation of our study and a larger sample size would definitely have made the results more relevant. Most patients of VCI also have significant motor disability which may influence the occurrence and/or severity of BPSD. Our study did not compare BPSD with the functional status of these patients or with respect to their age and gender. Moreover, there was a referral bias in our study because only patients with disabling strokes and significant motor deficits were referred to our institute. The motor symptoms overshadowed the cognitive and behavioural symptoms as the presenting complaint by most families. Contrary to the literature, we had fewer cases of SIVD, probably because these patients tended to have fewer disabling motor deficits and hence were not referred. We also observed a tendency on the part of many families to attribute cognitive and behavioural disturbances to age, socioeconomic problems, or supernatural factors and to delay seeking medical help for these problems until they became unmanageable.

In conclusion, BPSD are very common in poststroke VCI. Depression was found to be the commonest symptom, irrespective of the subtype or severity of VCI. Apathy was both commoner and more severe in SIVD than in the multi-infarct dementia group. The total NPI score was also significantly higher in the former group. Otherwise, these two subgroups were comparable. Apathy and appetite disturbances showed an increase with increasing CDR scores. The severity of apathy, appetite, and night-time behaviour disturbances as well as the total NPI scores correlated positively with worsening degrees of cognitive impairment.

Early characterisation and management of BPSD in VCI are important inasmuch as the alleviation of these distressing symptoms can improve the performance of these patients and ease the burden of their caregivers. Further elucidation of the anatomical basis of various neuropsychiatric symptoms especially in strategic infarct dementia has a potential to throw more light on the biology of BPSD and to help develop more effective treatment modalities for these conditions.

## Figures and Tables

**Figure 1:**
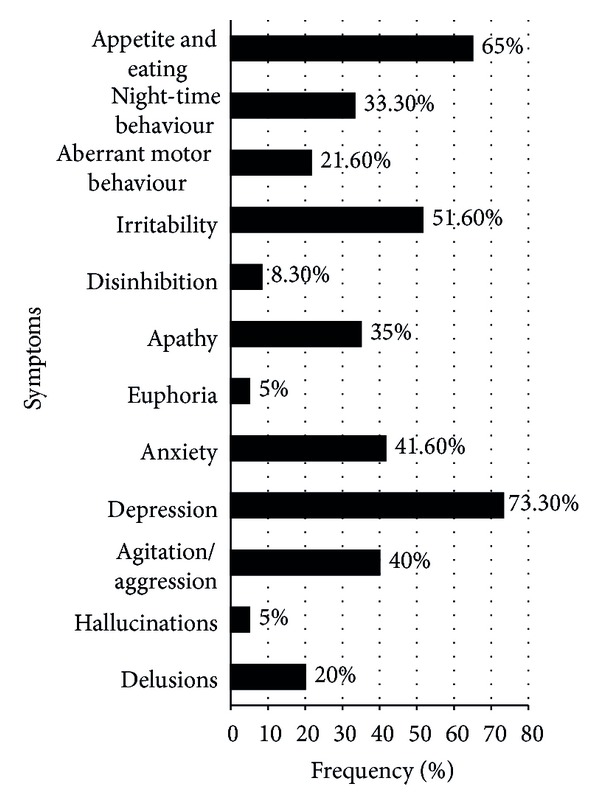
Percentage frequency of various behavioural and psychological symptoms.

**Table 1:** Demographic characteristics of the sample.

Parameter	MID	SIVD	Strategic	*P* value	Total
Mean age (SD)	58.32 (12.82)	62.57 (8.91)	54 (13.53)	0.114*	58.8 (12.03)
Number (%) of males	18 (72)	12 (57.14)	10 (71.4)	0.51**	40 (66.66)
Mean (SD) years of education	7.4 (4.48)	7.23 (4.96)	9.85 (6.29)	0.275*	8 (5.14)
CDR: 0.5/1/2/3 (*n* = )	7/6/6/6	6/5/4/6	13/1/0/0	0.004**; (0.97 (MID versus SIVD))	26/12/10/12

*Analysis of variance; **Chi-square test.

**Table 2:** Frequency of BPSD in the various subtypes of VCI.

Symptoms	MID *N* (%)	SIVD *N* (%)	Strategic *N* (%)	Total *N* (%)	*P* value MID versus SIVD*
Delusions	4 (16.00)	7 (33.33)	1 (7.14)	12 (20)	0.17
Hallucinations	2 (8.00)	1 (4.76)	0	3 (5)	0.196
Agitation/aggression	13 (52.00)	10 (47.62)	1 (7.14)	24 (40)	0.767
Depression	18 (72.00)	16 (76.19)	10 (71.43)	44 (73.3)	0.747
Anxiety	9 (36.00)	12 (57.14)	4 (28.57)	25 (41.6)	0.152
Euphoria	3 (12.00)	0	0	3 (5)	0.101
Apathy	7 (28.00)	13 (61.90)	1 (7.14)	21 (35)	**0.021**
Disinhibition	2 (8.00)	3 (14.29)	0	5 (8.3)	0.495
Irritability	18 (72.00)	11 (52.38)	2 (14.29)	31 (51.6)	0.17
Aberrant motor behaviour	5 (20.00)	8 (38.10)	0	13 (21.6)	0.175
Night-time behaviour	8 (32.00)	11 (52.38)	1 (7.14)	20 (33.3)	0.162
Appetite and eating	17 (68.00)	17 (80.95)	5 (35.71)	39 (65)	0.319
Mean (SD) number of symptoms**	4.24 (1.61)	5.14 (1.85)	1.78 (1.62)	3.98 (2.11)	0.08**

*Chi-square test.

**Unpaired *t*-test.

## References

[1] Rockwell E, Jackson E, Vilke G, Jeste DV (1994). A study of delusions in a large cohort of Alzheimer’s disease patients. *The American Journal of Geriatric Psychiatry*.

[2] Tun S-M, Murman DL, Long HL, Colenda CC, von Eye A (2007). Predictive validity of neuropsychiatric subgroups on nursing home placement and survival in patients with Alzheimer disease. *The American Journal of Geriatric Psychiatry*.

[3] Miyamoto Y, Tachimori H, Ito H (2010). Formal caregiver burden in dementia: impact of behavioral and psychological symptoms of dementia and activities of daily living. *Geriatric Nursing*.

[4] Hachinski VC, Iliff LD, Zilhka E (1975). Cerebral blood flow in dementia. *Archives of Neurology*.

[5] Roman GC, Tatemichi TK, Erkinjuntti T (1993). Vascular dementia: diagnostic criteria for research studies: report of the NINDS-AIREN International Workshop. *Neurology*.

[6] Ingles JL, Wentzel C, Fisk JD, Rockwood K (2002). Neuropsychological predictors of incident dementia in patients with vascular cognitive impairment, without dementia. *Stroke*.

[7] Hughes CP, Berg L, Danziger WL, Coben LA, Martin RL (1982). A new clinical scale for the staging of dementia. *British Journal of Psychiatry*.

[8] Cummings JL, Mega M, Gray K, Rosenberg-Thompson S, Carusi DA, Gornbein J (1994). The neuropsychiatric inventory: comprehensive assessment of psychopathology in dementia. *Neurology*.

[9] Staekenborg SS, Su T, van Straaten ECW (2010). Behavioural and psychological symptoms in vascular dementia; differences between small- and large-vessel disease. *Journal of Neurology, Neurosurgery and Psychiatry*.

[10] Chiu P-Y, Liu C-H, Tsai C-H (2007). Neuropsychiatric manifestations in vascular cognitive impairment patients with and without dementia. *Acta Neurologica Taiwanica*.

[11] Srikanth S, Nagaraja AV, Ratnavalli E (2005). Neuropsychiatric symptoms in dementia-frequency, relationship to dementia severity and comparison in Alzheimer’s disease, vascular dementia and frontotemporal dementia. *Journal of the Neurological Sciences*.

[12] Fuh J-L, Wang S-J, Cummings JL (2005). Neuropsychiatric profiles in patients with Alzheimer’s disease and vascular dementia. *Journal of Neurology, Neurosurgery and Psychiatry*.

[13] Johnson DK, Watts AS, Chapin BA, Anderson R, Burns JM (2011). Neuropsychiatric profiles in dementia. *Alzheimer Disease and Associated Disorders*.

[14] Kim JS, Choi S, Kwon SU, Seo YS (2002). Inability to control anger or aggression after stroke. *Neurology*.

[15] Aalten P, Verhey FRJ, Boziki M (2007). Consistency of neuropsychiatric syndromes across dementias: results from the European Alzheimer Disease Consortium. *Dementia and Geriatric Cognitive Disorders*.

[16] Kim J-K, Lyons D, Shin I-S, Yoon J-S (2003). Differences in the behavioral and psychological symptoms between Alzheimer’s disease and vascular dementia: are the different pharmacologic treatment strategies justifiable?. *Human Psychopharmacology*.

[17] Aharon-Peretz J, Kliot D, Tomer R (2000). Behavioral differences between white matter lacunar dementia and Alzheimer’s disease: a comparison on the neuropsychiatric inventory. *Dementia and Geriatric Cognitive Disorders*.

